# Emerging targets in cancer drug resistance

**DOI:** 10.20517/cdr.2018.27

**Published:** 2019-06-19

**Authors:** Shashank Kumar, Prem Prakash Kushwaha, Sanjay Gupta

**Affiliations:** ^1^School of Basic and Applied Sciences, Department of Biochemistry and Microbial Sciences, Central University of Punjab, Bathinda 151001, India.; ^2^Department of Urology, Case Western Reserve University, Cleveland, Ohio 44106, USA.; ^3^The Urology Institute, University Hospitals Cleveland Medical Center, Cleveland, Ohio 44106, USA.; ^4^Department of Nutrition, Case Western Reserve University, Cleveland, Ohio 44106, USA.; ^5^Divison of General Medical Sciences, Case Comprehensive Cancer Center, Cleveland, Ohio 44106, USA.; ^6^Department of Urology, Louis Stokes Cleveland Veterans Affairs Medical Center, Cleveland, Ohio 44106, USA.

**Keywords:** Drug resistance, transforming growth factor-β, Keap1-Nrf2, PI3K-Akt, FOXO transcription factors, focal adhesion kinases, annexins, MIEN1, gene splicing, sphingolipids, microRNA

## Abstract

Drug resistance is a complex phenomenon that frequently develops as a failure to chemotherapy during cancer treatment. Malignant cells increasingly generate resistance to various chemotherapeutic drugs through distinct mechanisms and pathways. Understanding the molecular mechanisms involved in drug resistance remains an important area of research for identification of precise targets and drug discovery to improve therapeutic outcomes. This review highlights the role of some recent emerging targets and pathways which play critical role in driving drug resistance.

## Introduction

Drug resistance during cancer treatment frequently originates with the failure of chemotherapy. The term “chemotherapy” was first introduced 70 years back by Goodman and co-workers for the treatment of leukemia and lymphosarcoma at the end of the II World war^[1]^. Since then chemotherapy remains a mainstay treatment modality in cancer management. A large number of targets and treatment approaches to cancer have recently emerged, however pertinent resistance and severe side-effects remains a major clinical problem. A large patient inter-individual variability in their pharmacokinetics and inconsistent antitumor effects has been observed for most anticancer drugs^[2]^. However, most patients do not respond to these drugs and they often experience severe adverse effects such as neutropenia, diarrhea and potential hair loss. The major reason for this effect remains indiscriminate targeting of both normal and malignant cells by these chemotherapeutic agents. More than 200 anticancer drugs including cytotoxic and biologically targeted agents are currently in use having a low success rate of ~5% in the clinical application^[3]^. Therefore, identification of novel drug targets and development of effective chemotherapeutic agents to overcome drug resistance in cancer remains a major priority. Based on tumor response to the initial therapy, chemo-resistance mechanisms are classified into two categories including *de novo* (intrinsic) and acquired (extrinsic), however, the detailed mechanism(s) of chemo-resistance in human cancers remains to be understood completely.

Previous studies have identified drug efflux transporters/multi drug resistance pumps, uridine diphospho-glucuronosyltransferase (UGT) superfamily, cytochrome P450s and *Glutathione S-transferases* (GSTs) as some classical drug resistance target(s) in cancer chemotherapy^[4-6]^. GSTs includes eukaryotic and prokaryotic phase II family isozyme. For the detoxification process, GST catalyzes the reduced form of glutathione (GSH) to xenobiotic substrates. GST expression levels are linked with higher tumor drug resistance. Another GST specific isoenzyme called Glutathione S-transferases P are ubiquitous in nature and showed elevated levels in non-drug resistant and drug-resistant cancers^[7]^. ATP-binding cassette (ABC) transporters are the membrane-bound proteins require ATP for functioning. These transporters allow the substrates in (influx) or out (efflux) of the cells. Higher expression of ABC transporter also results in efflux of cytotoxic agent from the cancer cells which leads to drug resistance. In addition to above mechanisms of drug resistance, mutation-induced alterations in cytochrome P450 activity or other types of modifications in malignant cells including glycosylation of anticancer agents by UGT superfamily may modify drug efficacy as a result of altered drug metabolism^[8]^.

More recent studies have identified abrupt cellular signaling pathways as underlying mechanisms for the development of cancer drug resistance. Some key process involve in drug resistance include tumor heterogeneity, reactivation of drug targets, hyperactivation of alternative pathways, cross-talk with the microenvironment, altered DNA response and its repair, modification in epigenetic pathways, impairment in apoptosis/autophagy and existence of cancer stem cells^[9]^. Any alteration in these pathways initially initiates changes in metabolic pathways and alteration in endocrine system functioning with later association with uncontrolled cell division through gene regulation. Innovations in molecular and biochemical techniques led to the identification/establishment of some new pathways including transforming growth factor beta (TGF-β) and Keap1-Nrf2 and some emerging drug resistance targets such as FOXO3A, FOXM1, FAKs, ANXA2, KCNN3, MIEN1 and epigenetic modifiers such as miRNAs and alternative splicing. In the present review we discussed these novel anticancer drug resistant targets and pathways.

## Drug transport and metabolism

Various trans-membranous proteins such as ABC have been well reported for the resistance initiation against several chemotherapeutic drugs. These include P-glycoprotein known as multi-drug resistance protein 1 (MDR1), ABCC1 known as MDR-associated protein 1 (MRP1) and ABCG2 known as breast cancer resistance protein (BCRP). These transporter proteins are highly specific to eliminate cancer therapeutic drugs such as topoisomerase inhibitors, taxanes, and antimetabolites. Epithelial cells which participate in the excretion process express higher levels of MDR1 proteins^[10]^. Several studies demonstrate that overexpression of MDR1 in different cancers such as colorectal, hepatocellular, renal, breast, lung, prostate, lymphomas and leukemia’s have been tightly associated with chemo-resistance^[11-14]^. Breast cancer resistance protein also possess chemo-resistive properties against leukemia^[15,16]^. Some cancer therapeutic drugs such as nilotinib, erlotinib, sunitinib, and imatinib are prime target for MDR1 and BCRP efflux pumps^[17]^. MDR1 pump inhibitor, tariquidar in combination with anthracycline/taxanes showed partial activity in stage III/IV breast carcinoma during clinical trials^[18]^. In addition to this, another MDR1 inhibitor zosuquidar in combination with docetaxel did not show any effect in overall survival^[19]^. These facts suggest that the ABC transporter family comprise a high degree of functional redundancy.

Metabolism of chemotherapeutic drugs also possesses a major role in drug resistance initiation. Several studies have shown that activation or inactivation of chemotherapeutic drugs leads to drug resistance. Meijer *et al*.^[20]^ demonstrated that the thiol group of glutathione inactivates platinum-based chemotherapeutic drugs. Absence or inactive form of the cellular enzyme that converts methotrexate and 5-fluorouracil (5-FU) to their active forms are also responsible for drug resistance^[21,22]^. Thymidine phosphorylase converts fluoropyrimidine prodrug (capecitabine) to 5-FU^[23]^. Methylation of thymidine phosphorylase encoding gene can cause capecitabine resistance^[24]^. UDP glucuronosyltransferase 1 promoter methylation inactivates its expression which positively regulates topoisomerase I inhibitor irinotecan drug and protects it’s from deactivation^[25,26]^.

## Cellilar signaling pathways in drug resistance

**Figure 1:**
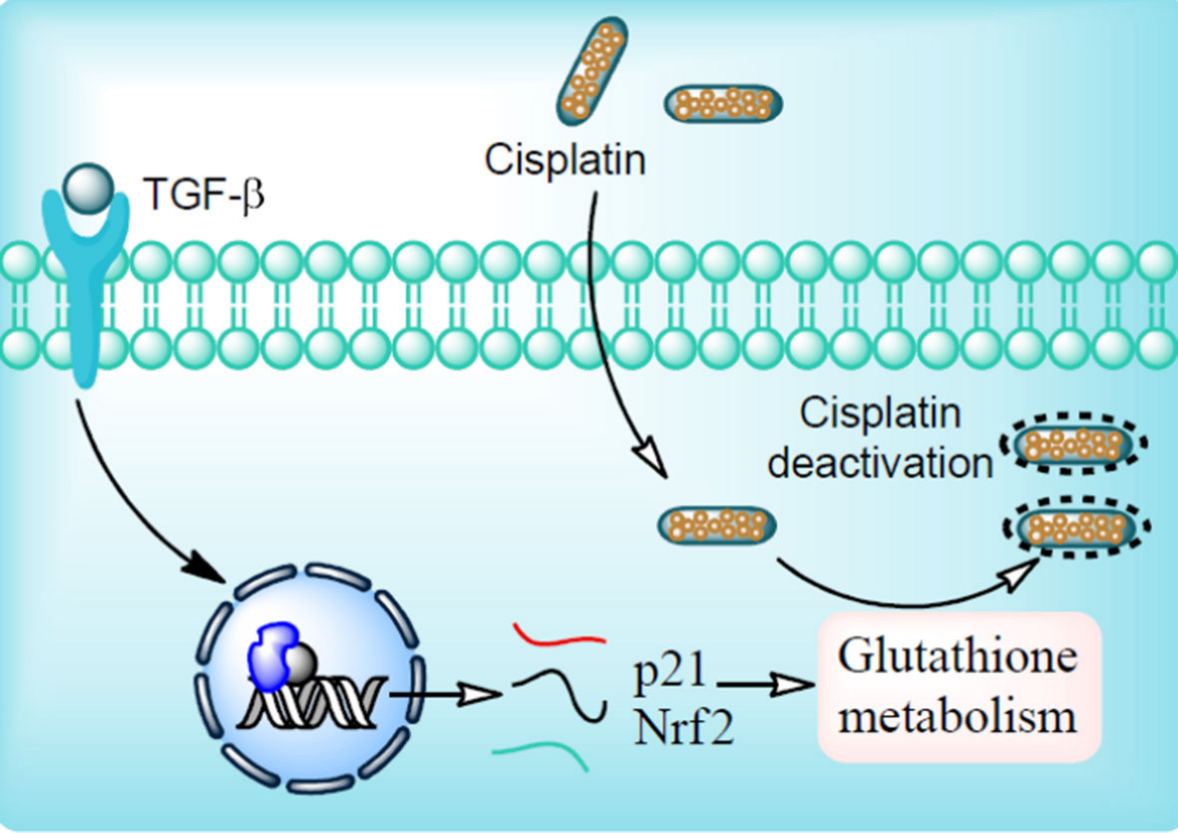
Mechanism of cisplatin resistance. Ligand binding to transforming growth factor beta (TGF-β) receptor initiates intracellular signaling through Smad protein complex (SPC). In the nucleus, SPC bind with the DNA binding domain which results in expression of *p21/Waf1* and *Nrf2* gene. *p21/Waf1* and *Nrf2* gene products tightly regulate glutathione metabolism. Cisplatin enters cells by passive diffusion. At low chloride ion concentration, the chloride ion of cisplatin is replaced with water molecules and forms activated cisplatin (aquation). Activated cisplatin enters the nucleus and results into the transcription of genes involved in anticancer activity. Glutathione conjugation with cisplatin hinders its nuclear translocation and thereby its chemo-preventive potential resulting into cisplatin resistance

The TGF-β pathway regulates numerous cellular processes including cellular growth and differentiation, apoptosis and maintains cellular homeostasis^[27]^. TGF-β plays an important role in the maintenance of tumor microenvironment and its subsequent progression. Both intracellular and extracellular signals (DNA damage and TGF-β) have potential to induce p21/Waf1 gene transcription. Oxidative stress also elevates the p21/Waf1 and Nrf2 gene expression. Together, these proteins remarkably enhance the glutathione metabolism and reduce the potential of the anticancer drug such as cisplatin^[28]^ [Figure 1].

**Figure 2:**
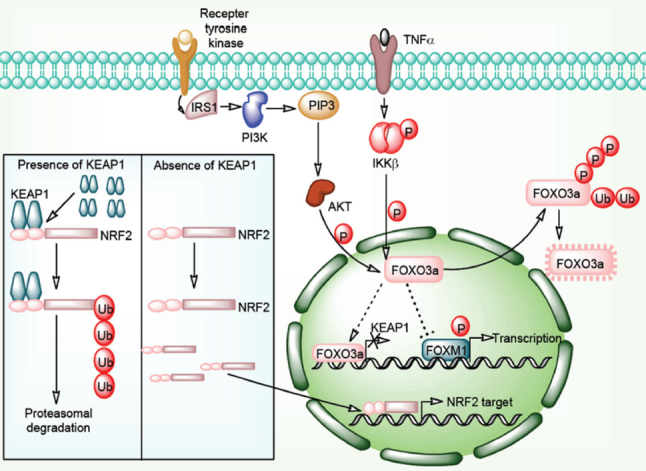
Keap1-Nrf2 signaling pathway in cancer drug resistance. Interaction of Keap1 molecules to Nrf2 protein is followed by Cul3-based E3 ligase complex mediated Nrf2 polyubiquitylation results into its proteasomal degradation. In the absence of Keap1 molecules, Nrf2 freely enters in the nucleus and transcribes its target genes in association with other nuclear factors. PI3K/Akt/TNF-α/NF-κB pathway directly phosphorylates FOXO3a proteins and directs them for ubiquitination and proteasomal degradation. Normally, FOXO3a proteins inhibits FOXM1 function and represses FOXM1 targeted transcription. FOXO3a protein also transcribes *Keap1* genes. Absence of FOXO3a protein results in to downregulation of *Keap1* *mRNA* and *FOXM1* targeted genes

The Cap’n’collar (CNC) family proteins play important role in gene transcription and mammalian developmental process. Nuclear factor erythroid 2-related factor 2 (Nrf2) is an important member of CNC family proteins^[29]^. Nrf2 is a transcription factor known to modulate the expression of genes involved in the cellular antioxidant-oxidant system. Kelch-like ECH-associated protein 1 (Keap1) is an E3 ubiquitn ligase adaptor protein responsible for its interaction with the Nrf2 protein. Double glycine repeat (DGR) and c-terminal region of Keap1 interacts with the motifs of Nrf2. This interaction results in the proteasomal degradation of Nrf2 transcription factor in a constitutive manner. Oxidative stress weakens the Keap1-Nrf2 interaction resulting into the dissociation and translocation of Nrf2 into the nucleus. In the nucleus Nrf2 induces the transcription of genes involved in cytoprotection and metabolic pathways. In malignant cells, constitutive degradation of Nrf2 is disrupted which results in the increase in Nrf2 responsive gene expression. Keap1 and Nrf2 gene mutations, exon loss in Nrf2 gene, methylation of Keap1 promoter, sequestosome 1 protein accumulation, fumarate hydratase mutation, and activation of Nrf2 gene are some critical factors involved in the Nrf2 induction^[30]^ [Figure 2].

Apart from the TGF-β and Keap1-Nrf2 signaling, mutation in the drug transporter gene could lead to drug resistance. Cisplatin uptake transporter named as CTR1 (*SLC31A1*) can be regulated by mutations which result in the introduction of drug resistance^[31]^. Mutation in the reduced folate carrier (*RFC1/SLC19A1*) protein causes methotrexate (MTX) drug resistance^[32]^. This could be achieved by efflux of intracellular MTX by MRP1 drug efflux pumps. Alteration in the MTX polyglutamylation also diminishes the MTX cellular sensitivity. MTX polyglutamates can also prevent dihydrofolate reductase (DHFR) enzyme activity inhibiting the conversion of dihydrofolate (FH_2_) to tetrahydrofolate (FH_4_)^[33]^. Absence of tetrahydrofolate reduces DNA synthesis and induces cell death. Mutation in DHFR enzyme induces obstruction between the interaction of MTX and its polyglutamates. MTX and its polyglutamates suppresses thymidylate synthesis which also diminishes DNA synthesis. Alterations in the levels or affinity of these enzymes in the cellular system develop the drug resistance. Drug resistance can also be developed by high copy number of the DHFR gene expression^[34]^.

Inside the cell, water replaces the Cl groups of cisplatin and generates reactive nucleophilic species. These species have the ability to penetrate the nuclear membrane and can form covalent bond with the DNA molecule (primarily guanine) which initiates the process of cell death. Activation of nucleotide excision repair or other repair pathway introduces resistance in cancer cells. Prior to cisplatin entering in the nucleus, glutathione conjugation with cisplatin by the enzyme GSH S-transferases reduces drug effectiveness^[35]^. Cisplatin interaction with the sulfhydryl-containing metallothioneins also diminishes drug efficacy. Alternative pathway of cellular cisplatin resistance includes the efflux activity of the MRP2 (ABCC2) transporter and ATP7B P-type adenosine triphosphatase (ATPase) transporter^[36]^.

**Figure 3:**
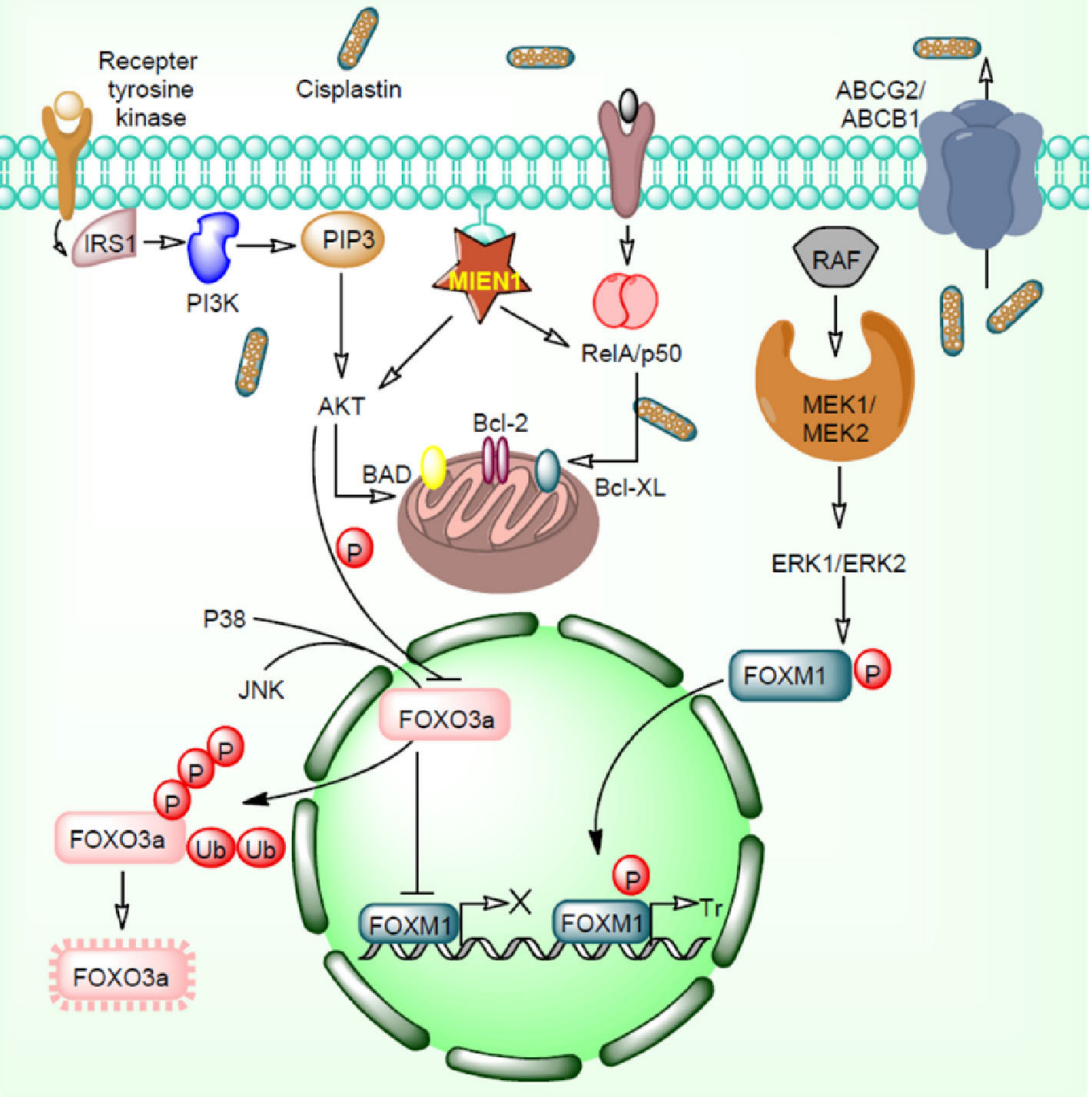
EGFR/PI3K/MAPK/ERK1/2-FOXO3a pathways in cancer drug resistance. Overexpression of MIEN1 and ABCG2/ABCB1 initiates the cisplatin resistance by targeting Akt/RelA/p50 and efflux of cisplatin respectively. MIEN1 targets Akt/RelA/p50 and induces overexpression of the anti-apoptotic proteins. Akt protein activated by MIEN1 inhibits FOXO3a function. Together, P38 and JNK phosphorylate FOXO3a protein which results in to its ubiquitination and proteasomal degradation. MEK/ERK pathway phosphorylates FOXM1 proteins which results in to translocation of these proteins inside the nucleus. Phosphorylated FOXM1 transcribes several genes which positively involves in drug resistance

The EGFR/PI3K-PTEN/Akt/mTOR/ and Wnt/β-catenin signaling pathways play important roles in the malignant transformation and drug resistance^[37]^. These signaling pathways are frequently altered in various cancers due to the anomalous expression levels of PTEN, HER2, EGFR1, and other tumor suppressor gene/oncogenes. Some of the kinases involved in the cellular signaling also play a comprehensive role in cancer development and drug resistance establishment^[38]^. Due to its substantial implication in drug resistance development, kinases are the second largest drug target family. In addition to this, tumor microenvironment positively regulates these kinases and helps tumor to acquire resistance properties. Several signaling pathways have been reported for their outstanding contribution to the maintenance of tumor microenvironment. Mutation in EGFR and its downstream signaling leads to the expansion of cancer cell population^[39]^. Another transcription factor, polyoma enhancer activator 3 (Pea3) has been reported for its pivotal engrossment in invasion and metastasis in small cell lung cancer. Vascular endothelial growth factor stimulates angiogenesis and vasculogenesis (blood vessel formation) having potential to induce drug resistance. Another well-known signaling molecule Wnt16B regulates important factor required for cell proliferation and epithelial-mesenchymal transition in cancer cells^[40]^. Nuclear factor-κB (NF-κB) regulates the Wnt16B expression levels during these events. These pathways create a boundary and limit the drug efficacy and lead to development of drug resistance. Additionally, malignant cells secrete plasminogen activator inhibitor-1 which provides aggressiveness and facilitates cell proliferation^[41]^. AKT and ERK1/2 signaling and reduced level of caspase 3 participate in these events [Figure 3].

## Genetic modifications in drug resistance

### DNA mutation

Variation in expression or mutation in the drug target proteins may be the major reason for drug resistance initiation. Fluorodeoxyuridine monophosphate (FdUMP) actively targets thymidyate synthase^[42]^. One of the studies demonstrated that thymidyate synthase expression determines the 5-FU sensitivity^[43]^. Thymidyate synthase promoter polymorphism (TSER3/TSER3) negatively regulates 5-FU-based chemotherapy than the heterozygous (TSER2/TSER3) /homozygous alternative polymorphism (TSER2/TSER2)^[44]^. Overexpression of thymidyate synthase encourages resistance to its inhibitors like TDX, 5-FU, and multi-targeted anti-folate^[45,46]^. SN-38, a metabolite of CPT-11, inhibits DNA topoisomerase-I enzyme which relaxes super-coiled double-stranded DNA during the replication process^[47]^. Downregulation of topoisomerase-I mRNA results in anti-sensitivity against CPT-11 in colorectal cancer^[48]^. Anthracyclines (doxorubicin) and epipodophyllotoxins (etoposide) are ideal target for the topoisomerase-II enzyme which also plays a vital role during replication. Alteration in topoisomerase-II expression levels or mutation in this protein results in resistant to doxorubicin/etoposide in colon cancer cells^[49,50]^.

Tubulin proteins such as α- and β-tubulin generate microtubule structures that maintain cell integrity, regulates signaling/cell division and helps in vesicle transportation throughout the cellular compartments^[51]^. Tubulin polymerization/de-polymerization regulates appropriate spindle formation and chromosome segregation during cell division. Taxanes (paclitaxel and docetaxel) and vinca alkaloids (vinblastine and vincristine) repress the polymerization of tubulin which blocks cell division^[52,53]^. An *in vitro* study reported that mutation in the β-tubulin proteins regulates the sensitivity against paclitaxel^[54,55]^.

### DNA repair

Mutation in gene encoding enzymes involved in DNA damage response (DDR) such as ataxia telangiectasia (ATM), ATR, RAD50, and WRN can enhance the risk of cancer emergence and resistance^[56]^. Single nucleotide polymorphisms in DNA repair-associated genes can also induce cancer drug resistance^[57]^. Some of the chemotherapeutic drugs such as 5-fluorouracil, epirubicin, cisplatin, and doxorubicin have been well studied in cancer for their potential to cause damage in the DNA molecule. DNA damage triggers DDR proteins to repair the DNA and induces drug resistance in cancer cells^[58]^. DNA repair associated (DRCA) genes directly regulate DNA repair pathway via the homologous recombination and DNA strand breaks. Development of inhibitors for these proteins are directly involved in the repair pathway can reduce drug resistance and sensitize to these agents. Poly ADP-ribose polymerase 1 (PARP1) is an important protein which modified nuclear proteins through the poly ADP-ribosylation and makes them active for the DNA damage repair. Olaparib, USFDA approved drug, is a PARP inhibitor which blocks DNA repair functions and induces apoptosis in cancer cells^[58]^. Another DNA repair gene namely NEK2, codes serine/threonine mitotic kinase plays an important role in spindle formation and chromosome segregation^[59,60]^. Silencing of NEK2 reduces drug resistance to bortezomib *in vitro* and *in vivo*. NER pathway comprises excision repair cross-complementing 1, whose increased levels has been linked to poor response to chemotherapy in ovarian, gastric and non-small-cell lung carcinoma^[61,62]^. Cisplatin-treated testicular cancers showed decreased levels of excision repair cross-complementing 1^[63]^.

## Epigenetic modifications in drug resistance

### Alternative splicing

Splicing events after the post transcription process matures the mRNA to become respective functional protein. Any deformities in these processes produce nonfunctional or mutated proteins resulting in cancer initiation and development. Alternative splicing accomplishes coding region reshuffling and regulates mRNA maturation^[64]^. Defects in splicing mechanism lead to drug resistance against selected anticancer agents. BIM, a type of BH3-only proteins belongs to the pro-apoptotic B-cell CLL/lymphoma 2 (BCL-2) protein family. It play critical role in hematopoietic homeostasis, autoimmune disease restriction and cancer initiation. BimEL, BimL and BimS are the three major BIM alternative splicing products^[65]^. Studies provide evidence that BIM alternative splicing molecules play a key role in drug resistance^[66]^. Upregulated BIM proteins facilitate tyrosine kinase inhibitors stimulated cell death events in kinase dependent tumors. Polymorphism also play a critical role in the origination of drug resistance. For example, an intron comprising 2903-bp deletion in BIM gene recruits privileged splicing of exon 3 over exon 4 produces BIM isoforms which does not bears BCL-2 homology domain 3 (BH3) domains. This type of BIM protein promotes drug resistance in NSCLC (non-small cell lung cancer) and CML (chronic myeloid leukemia) against tyrosine kinase inhibitors such as gefitinib and imatinib^[67]^.

Folylpolyglutamate synthase (FPGS) enzyme is involved in the maintenance of folate homeostasis in the cytosolic and mitochondrial compartment. Exon 12 skipping in FPGS gene results in dysfunctional FPGS which leads to the inhibition of anti-folate drugs such as methotrexate and introduces resistance in acute lymphoblastic leukemia^[68]^.

CART-19 immunotherapy is well known to recognize the full length CD19 protein which diminishes the death probability of cancer cells. Skipping of exon 2, in the maturation of CD19 mRNA, results in the truncated CD19 protein. This splicing event reduces the probability of tumor aggressiveness identification at the early stage and induces drug resistance^[69]^. In BRCA1 gene, skipping of exon 11 restores BRCA1 DNA repair potential and stimulates drug resistance^[70]^.

### DNA modification

Epigenetic modifications play a significant role in the expression of various receptor proteins such as hormone receptors. Tamoxifen treatment downregulates estrogen receptor expression after treatment in estrogen-responsive breast cancer. This may occur due to epigenetic silencing of the estrogen receptor-encoding genes. A study has shown that aberrant methylation of CpG islands of the estrogen receptor promoter and histone deacetylation downregulates its expression and induces tamoxifen resistance. Overexpression of HER2/EGFR, hypoxia and MAPKs hyper-activation downregulates the estrogen receptor expression. MiRNA alteration has been directly associated with tamoxifen resistance in breast cancer^[71]^. Steroid receptor coactivator-3 (SRC-3) regulates various processes of cancer development and act as a coactivator/transcription factor. Breast cancer cells have been reported for the overexpression of SRC-3 and positively correlated with tamoxifen resistance^[72]^. SRC-3 over-expression is also positively correlated with increased levels of HER-2/neu, poor disease-free survival and tamoxifen resistance in tamoxifen-treated breast cancer cells^[73]^. Epigenetic-driven drug resistance includes epigenetic modification in the gene of pro-apoptotic (DAPK, APAF-1), drug transporters (ABCB1), histone modifiers (KDM5A) and DNA repair proteins (MLH1, MGMT, FANCF). Chemotherapeutic drug treatment in drug-resistant cancer cells alters various enzymes (involved in genome regulation) which makes drug-resistant epigenome to drug-sensitive epigenome thereby rendering cancer cells sensitive to therapeutic drugs.

### MicroRNA

Small noncoding RNA have been reported to control cell activity by regulating large number of target genes^[74]^. Recently researchers are focusing to acquire miRNA beneficial function with an attempt to overcome multidrug resistance-related phenomenon. MicroRNAs (miRNAs) are about 22-nucleotide long RNA produced from the proper processing of RNA structure. MiRNAs play a significant role in the regulation of gene expression^[75]^. It instructs numerous protein-coding genes including genes involved in cancer as well as drug resistance. Gene silencing involves the destruction of messenger RNA strand (mRNA) into two separate fragments. Gene silencing can also be achieved by diminishing the mRNA stability via shortening of poly (A) tail^[76]^. Another silencing mechanism included the reduced translation through ribosome of mRNA into proteins. Recent investigations have demonstrated that microRNAs play an important role in the development of drug resistance [Table 1]. MicroRNAs can assist as a biomarker for the patient survival in respect to drug-resistant therapies.

**Table 1:** miRNAs involved in chemo-resistance in various cancer and their targets

Chemotherapy agent	Target	Tumor	miRNAs	Ref.
Anthracyclines	MDR1	SCLC	miR-7	[77]
Temozolomide	MDR1/ABCG2	Glioblastoma	miR-9	[78]
Paclitaxel	PTEN, p-gp/ABCB1	Ovary, NSCLC	miR-17-5p, miR-145, miR-181a	[79-81]
Trastuzumab	PTEN, PDCD4	Breast	miR-21	[82]
Epriubicin	ABCG2	Breast	miR-25	[83]
Doxorubicin	P-gp, MRP1/ABCC1	Gastric, Brest	miR-103/107, miR-134	[84,85]
Adriamycin	MDR1/MRP1	Glioma	miR-127	[86]
Vincristine/cisplatin	ABCB1	Gastric	miR-129-5p	[87]
5-fluorouracil	ABCB1, P-gp/ABCB1, ABCG2	Gastric, Colorectal	miR-129-5p, miR-508-5p, miR-519c	[87-89]
Cisplatin	PTEN, MDR1/MRP1, Cyclin D1, GRB2, ERK2, RSK1, RSK2	NSCLC, Ovary	miR-181a, miR-196a, miR-634	[81,90,91]
5-fluorouracilmitomycin C	P-gp/ABCB1	Colorectal	miR-200c	[92]
Vincristine/oxaliplatin/cisplatin	P-gp/ABCB1	Colorectal, Gastric	miR-200c, miR-508-5p	[88,92]
Bortezomib	BAFF	Multiple myeloma	miR-202	[93]
Thalidomide	BAFF	Multiple myeloma	miR-202	[93]
Dexamethasone	BAFF	Multiple myeloma	miR-202	[93]
Tamoxifen	PTEN	Breast	miR-217	[94]
Lapatinib	PTEN	Breast	miR-217	[94]
Etoposide	PTEN	Breast	miR-217	[94]
Melphalan	MRP1/ABCC1	Multiple myeloma	miR-221/222	[95]
EGFR inhibitors	KRAS, AKT1	NSCLC		[96]

MDR: Multidrug resistance protein 1; SCLC: small cell lung cancer; ABCG2: ATP binding cassette subfamily G member 2; PTEN: phosphatase and tensin homolog; NSCLC: non-small cell lung cancer; p-gp: P-glycoprotein 1; ABCB1: ATP binding cassette subfamily B member 1; PDCD4: programmed cell death 4; MRP1: multidrug resistance-associated protein 1; ABCC1: ATP binding cassette subfamily C member 1; GRB2: growth factor receptor-bound protein 2; ERK2: extracellular signal-regulated kinase 2; RSK1: ribosomal protein S6 kinase A1; RSK2: ribosomal protein S6 kinase 2; BAFF: B-cell-activating factor of the tumor-necrosis-factor family; KRAS: Kirsten rat sarcoma 2; AKT1: AKT serine/threonine kinase 1; EGFR: epidermal growth factor receptor

## Novel targets in drug resistance

### FOXO3a and FOXM1

Numerous cytosolic, nuclear, intracellular and extracellular factors have been reported in cancer development and drug resistance^[97]^. These factors directly/indirectly regulate several others factors and cellular signaling pathways. Some factors have repressive or stimulatory potential to each other resulting into therapeutic drug resistance and increased aggressiveness in cancer cells. Transcription factors superfamily comprising DNA binding domain includes Forkhead box (FOX) proteins. FOX has been reported to regulate cellular machinery and various homeostatic processes. FOX factors controls numerous physiological processes including cell division, cell death, cell invasion and migration and drug resistance^[98-100]^. FOXO3a receives signals from the signaling pathways such as EGFR/PI3K/Akt/ERK, and transfers it to other pathway which ultimately controls transcription of respective gene. Sometimes these factors alone bind to the regulatory region of the promoter and thereby regulate gene transcription. Hyperactivation of PI3K/Akt signaling inactivates FOX factors and induces drug resistance. Mutation in the *PTEN* gene and receptor tyrosine kinases overexpression together endorses cancer development and drug resistance^[101]^. Several studies revealed that anticancer drugs such as imatinib, tamoxifen, cisplatin, doxorubicin, paclitaxel, lapatinib and gefitinib induce FOXO activation via alteration in PI3K/Akt signaling pathways^[99,102-105]^. JNK signaling also promotes FOXO3a nuclear localization and activity by diminishing Akt phosphorylation^[102,103]^ [Figure 3]. The p38 protein actively phosphorylates FOXO3a at Serine 7 residue which in turn enhances its nuclear re-localization and provides doxorubicin directed response^[98]^. Another FOX family factor, FOXM1 acts like an oncogenic transcription factor mainly controls cell cycle and division. FOXM1 is overexpressed in several cancers including breast, liver, colorectal, lung and the prostate^[106]^. Oncogenic potential of FOXM1 and its uncontrolled cell division properties makes it available in the stem cell compartments and initiate hyperplasia. FOXM1 imparts resistance against chemotherapeutic drugs such as epirubicin and cisplatin [Figure 3]. It also induces drug resistance via overexpression of DDR gene *viz*. replication factor C4 (RFC4), epsilon 2, accessory subunit (POLE2), polo-like kinase 4 (PLK4), X-ray repair complementing defective repair in Chinese hamster cells (XRCC1), breast cancer type 2 susceptibility protein (BRCA2), polymerase (DNA directed) and exonuclease 1 (EXO1)^[107-109]^. Furthermore, FOXM1 and FOXO3a together compete for the same DNA sequences for binding. These factors also share numerous target genes which facilitates FOXM1 transcriptional output repression by FOXO3a proteins. However, FOXO3a prevents VEGF expression and FOXM1 facilitates VEGF overexpression to regulate cell migration, invasion and drug resistance.

### Focal adhesion kinase

Focal adhesion kinase (FAK), a tyrosine kinase also referred as PTK2 (non-receptor protein tyrosine kinase) is a downstream protein of integrin and growth factor receptors signaling pathways. Increased expression of FAK has been associated with various type of cancer^[110]^. FAK modulates tumor progression, apoptosis, invasion, and metastasis. The N and C terminal domains (FERM and FAT domains) of the protein hinders the ATP binding site of the central kinase domain. The interaction between N and C terminal domain of the protein keeps it in an inactive form. Integrin and growth factor receptor activation induce disruption of N and C terminal domain resulting in the exposed ATP binding site on the central kinase domain. Binding of ATP to the central domain, phosphorylation of FAK-Y397 amino acid followed by SRC binding, and additional amino acid phosphorylation results in the activated FAK^[111]^. FAK induces PIP2/3 and AKT1-mediated survival signals in cells. FAK induces BCAR1 (breast cancer anti-estrogen resistance 1) and MAPK8 mediated cellular motility, proliferation, and survival in breast cancer. Beside FAK induces assembly and disassembly (turnover) of the focal adhesions and thus modulate migration of cells^[112]^. FAK is known to interact with some other proteins such as GRB2, TP53, MDM2, and RIP associated with the pathogenesis of cancer^[113]^. From the above discussion, it is clear that ATP binding domain and some other FAK domains involved in protein-protein interaction might serve as a cancer drug target. Inhibitors (VS-4718; VS-6062 and PF-573,228) having the binding ability to these domains serve as a promising candidate in anticancer drug discovery^[113]^. Studies indicate the promising efficacy of FAK inhibitors and chemotherapy synergism to reduce treatment side effects and drug resistance in cancer. These inhibitors increase chemosensitivity in drug-resistant cells and also synergize the drug treatment efficacy in cancer cells^[113]^. Recent reports suggest that FAK induces NF-κB pathway mediated cytokine production in response to DNA damage. This phenomenon protects the cells from DNA damage and maintains chemo-resistance in the cells^[114]^. In this regard, FAK inhibitors might play a critical role against DNA damage-mediated drug resistance in cancer cells.

### ANXA2

Annexins are multifunctional proteins having ability to bind phospholipids in the presence of calcium ions. It plays important role in cytoskeleton dynamics, signal transduction, and membrane trafficking. Due to these associated phenomenon annexins are involved in the pathophysiological condition of various diseases including cancer^[115]^. Annexin 2 has annexin core structure (highly conserved), N terminal domain (highly variable), calcium-dependent lipid binding region, RNA-binding helices, F-actin binding site and C terminal^[116]^. Phosphorylation of annexin 2 protein is essential for its multi-functions. In response to cell transformation, Src kinase phosphorylates annexin 2 (45 phosphorylation sites are known) mainly at the N terminal domain or the central domain^[116]^. Phosphorylation of Tyr23 residue in annexin 2 proteins has been associated with tumor cell adhesion, angiogenesis, invasion, motility, progression, and proliferation^[117]^. Annexin 2, Tyr23 phosphorylation mediated STAT3 phosphorylation is known to involve in glucocorticoid resistance in cancer cells^[118]^. Annexin 2 induce coilin disruption/chromosome instability mediated cellular chemo-resistance in cancer cells^[119]^. ANXA2 and tenascin-C protein interaction mediated activation of PI3K/Akt/NF-κB signaling pathway is involved in drug resistance in prostate cancer^[119]^. Moreover, annexin 2 is also known to involve in radiotherapy and immunotherapy in different cancer cells.

### Potassium calcium-activated channel subfamily N member 3

A potassium channel, potassium calcium-activated channel subfamily N member 3 (KCNN3) belongs to a trivial Ca^2+^-activated potassium channel family. KCNN3 have been reported for its role in solid tumor progression^[120]^. KCNN3 regulates cell membrane potential in melanoma and breast cancer cells^[120]^. Cell migration and invasiveness also partially regulated by P2X purinoceptor 7 and KCNN3. Transient receptor potential channel 1 and calcium release-activated calcium channel protein 1 with KCNN3 regulates store-operated calcium entry (SOCE)-dependent cell migration^[121]^. Upregulation of KCNN3 is positively associated with bortezomib-resistant myeloma cells which induce drug resistance^[120]^.

### Migration and invasion enhancer 1

Migration and invasion enhancer 1 (*MIEN1*) gene also known as C35, is a novel gene situated subsequent to the cluster of differentiation 340 (CD340) in the 17q12 amplicon of the humanoid chromosome^[122]^. The gene has unusual potential to increase tumor cell migration and invasion. MIEN1 expresses differentially in normal cells and cancer cells^[123]^. Regulation of MIEN1 by miRNAs may permit better targeting strategies to overcome the respective ailment. The hsa-miR-940 (miR-940) has been reported for their high expression levels in commemorated normal cells compared to tumor cells^[123]^. A study reported that hsa-miR-940 directly targets MIEN1 RNA and alters its downstream effectors in prostate cancer^[123]^. Inactivation of MIEN1 by miR-940 inhibits cell migration and invasion, diminished cell anchorage-independent growth aptitude and initiates overexpression of E-cadherin molecule which overcomes mesenchymal-to-epithelial transition (MET) process^[123]^. Another study reported that MIEN1, potassium calcium-activated channel subfamily N member 3 (KCNN3), and drug resistance in ovarian cancer are significantly associated with each other^[120]^. MIEN1 and DNp73 interaction induces chemo-resistance in ovarian cancer^[124]^. High levels of MIEN1 significantly promotes phosphorylation of tyrosine 23 (Y23) residue on annexin A2 (ANXA2) which facilitates the interaction with cellular actin filaments and regulates cell trickling/cytoskeletal rearrangement through actin transformation^[119]^. Earlier, it has been reported that ANXA2 expression correlates with poor prognosis and a higher number of chemotherapy cycles which stimulate chemoresistance^[125]^. Hypoxia and chemokines facilitate the transformation of human neural stem cells to glioma stem cells in presence of MIEN1^[126]^. FAK activating protein MIEN1 phosphorylates FAK at Y925 and induced phosphorylation of ERK1/2, Akt and NF-κB^[127]^ [Figure 3]. Previously it has been shown that FAK phosphorylation promotes overall survival of ovarian cancer patients^[128]^. MET process also promotes FAK phosphorylation and enhances taxane resistance in ovarian cancer^[128]^. High-mobility group box 2 (HMGB2) binding protein interacts with MIEN1 and enhances cancer progression and proliferation^[129]^. Silencing of HMGB2, an interacting partner MIEN1 sensitizes head and neck squamous cell carcinoma against cisplatin and 5-fluorouracil^[130]^. MIEN1 showed increased expression in lapatinib-sensitive breast cancer cells compared to lapatinib-resistant breast cancer cells^[131]^. Colony growth in soft agar, invasion into collagen matrix and formation of large acinar structures in three-dimensional cell cultures experiment demonstrated that increased MIEN1 expression is highly associated with cell transformation including epithelial to mesenchymal transition and reduced expression of E-cadherin and keratin-8^[132]^. This study also demonstrated that cell transformation was dependent on Syk kinases.

### Sphingolipids

Membrane lipids family comprises sphingolipids, a fatty acid derivative of sphingosine which constructs lipid bilayer structure and maintains their fluidity^[133]^. Sphingolipids includes sphingosine 1-phosphate (S1P), ceramide, glucosylceramide (GlcCer) and sphingosine that regulates various biological events such as proliferation, apoptosis, inflammation, senescence and cell migration in cancer cells^[133,134]^. Alteration of sphingolipid metabolism, as well as ceramide accumulation, is reported as a major factor for resistance development against chemotherapy in cancer cells^[135]^. Ceramide metabolism vastly produces GlcCer as a product due to higher glucosylceramide synthase (GCS) enzyme activity in tumor cells^[136]^. Studies have demonstrated that GCS overexpression and its activity, positively correlated with ABC transporter facilitating drug resistance^[137,138]^. GCS knockdown significantly reduces the MDR1 expression, a gene that encodes for ABC transporter protein^[137,138]^. ABC family transporters also transport sphingolipids, phospholipids, and glucosylceramide across the lipid bilayer.

Sphingosine kinase 1 (SK1) and S1P metabolism regulate drug sensitivity against cancer cells because overexpression of these molecules provides shelter to cancer cells from drug treatment. A study reported its increased levels in camptothecin resistant prostate cancer cells^[139]^. Another *in vivo* study demonstrated that SK1 and S1P inflection results in cisplatin sensitivity toward the cellular slime mold *Dictyostelium discoideum*, a powerful non-mammalian model for drug target discovery and resistance^[140]^. Baran *et al*.^[141]^ reported that imbalance between C18-ceramide and S1P is associated with SK1 overexpression and BCR-ABL upregulation which ultimately leads to imatinib resistance in human chronic myeloid leukemia K562 cells. Downregulation of SK1 levels enhanced the imatinib drug sensitivity and induces the apoptosis in these cells. Another *in vivo* study demonstrated that silencing of sphingosine 1 phosphate phosphatase 1 through miR-95, endorses S1P-dependent resistance to radiation in breast/prostate tumors^[142]^.

### Future perspectives

Drug resistance in cancer, either intrinsic or acquired, substantially reduces the efficacy of chemotherapeutic drugs with poor prognosis in cancer patients. To achieve higher likelihood of therapeutic success, a complete understanding of the mechanisms underlying chemo-resistance is needed. Recent development of high-throughput screening technologies has enhanced the identification of intrinsic and extrinsic cellular pathways that may be targeted to prevent or reverse drug resistance. Other evolving techniques including open reading frame screens, RNA interference, genome editing, and proteomics analysis of drug resistant cell lines and tissues will provide important information and identification of novel targets to overcome tumor drug resistance. In addition, smarter means to deliver anticancer drugs through targeted nanotechnology approach is being tested. This knowledge will be extremely helpful for the development of precision therapies based on the prediction of tumor cell response to the currently available chemotherapeutic agents and also the discovery of novel therapeutic strategies to treat cancer or reverse tumor chemo-resistance. Further work is required to determine which subset of cancer patients are suitable candidates for a particular multi-targeted therapy or combination regimen affecting multiple targets. Additional research or modeling is also needed to identify what combination of targets can be expected to optimize therapy for particular cancer types. Such new knowledge will be translated into the development of innovative cancer therapeutics to overcome drug resistance.

## Conclusion

Increased understanding of the mechanisms underlying cancer drug resistance suggests that an integrated approach to cancer therapy is needed for targeting multiple signaling pathways. Recent use of molecularly targeted agents to target multiple signaling pathways remains an important approach in cancer treatment, however, use of these targeted therapies is not without limitations. Further research is needed to identify approaches to repurpose drugs to optimize therapy for particular cancer types.

## Declarations

### Copyright

© The Author(s) 2019.
